# Rare Cause of Restrictive Cardiomyopathy: A Case Report of Löffler Endocarditis

**DOI:** 10.1155/cric/5307648

**Published:** 2026-08-17

**Authors:** Jose L. Diz Ferre, Edward G. Soltesz, Agostina Fava

**Affiliations:** ^1^ Kaufman Center for Heart Failure, Heart, Vascular and Thoracic Institute, Cleveland Clinic, Cleveland, Ohio, USA, clevelandclinic.org; ^2^ Section of Regional Cardiovascular Medicine, Heart, Vascular and Thoracic Institute, Cleveland Clinic, Cleveland, Ohio, USA, clevelandclinic.org

## Abstract

Löffler endocarditis, an idiopathic hypereosinophilic syndrome, can present symptoms of heart failure and peripheral eosinophilia. We present the case of a 46‐year‐old woman with fatigue, shortness of breath, and edema, who was found to have thrombocytopenia, elevated cardiac biomarkers, and hypereosinophilia (2.96 k/uL). Cardiac MRI revealed hypertrophic cardiomyopathy, right ventricular hypertrophy, and a restrictive filling pattern, raising suspicion for Löffler endocarditis. Despite initial corticosteroid and diuretic treatment, she was readmitted with persistent symptoms. This case highlights the diagnostic imaging and treatment challenges with corticosteroid therapy, to control eosinophilic infiltration and prevent irreversible cardiac damage. Löffler endocarditis should be considered in the imaging differential diagnosis of unexplained heart failure.

**Take-Home Messages**

• Löffler endocarditis can present with nonspecific symptoms, hypereosinophilia, and heart failure due to restrictive cardiomyopathy.
• Cardiac MRI imaging can inform the diagnosis of Löffler endocarditis when endomyocardial biopsy is unsuccessful or not available.
• Response to steroids is necessary for effective treatment to control eosinophilic infiltration and prevent further endomyocardial damage.

## 1. History of Presentation

A 46‐year‐old African American woman presented to the emergency department with fatigue, weakness, and shortness of breath, describing a viral syndrome‐like illness. She denied fever, dysuria, flank pain, nausea, or vomiting. On examination, she was tachycardic. Electrocardiogram (ECG) revealed findings concerning for non–ST‐elevation myocardial infarction. Laboratory results showed thrombocytopenia (platelets 8 k/uL), anemia (hemoglobin 10.4 g/dL), and an elevated cardiac troponin I of 8.8 ng/mL. A COVID‐19 test was negative.

## 2. Past Medical History

The patient was otherwise healthy, with a past medical history of nontoxic thyroid nodule, hyperlipidemia, thrombocytopenia, hiatal hernia, gastroesophageal reflux disease, and a history of breast surgery. Her medications included an oral contraceptive.

## 3. Differential Diagnosis

Differential diagnosis included non–ST‐elevation myocardial infarction, endocarditis, myocarditis, and the most common infiltrative cardiomyopathies such as amyloidosis as well as primary myeloid disorder.

## 4. Investigations

**Figure 1:**
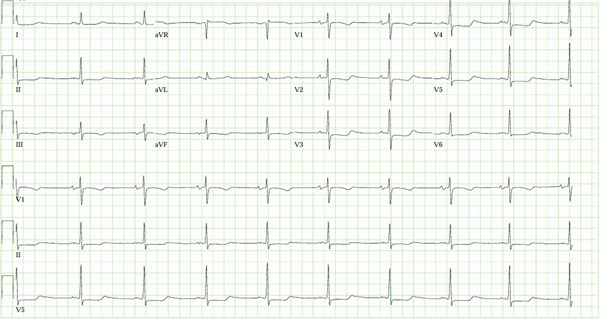
Electrocardiogram showing sinus bradycardia, anterior ST segment, and T wave abnormalities with prolonged QT interval or TU fusion.

**Figure 2:**
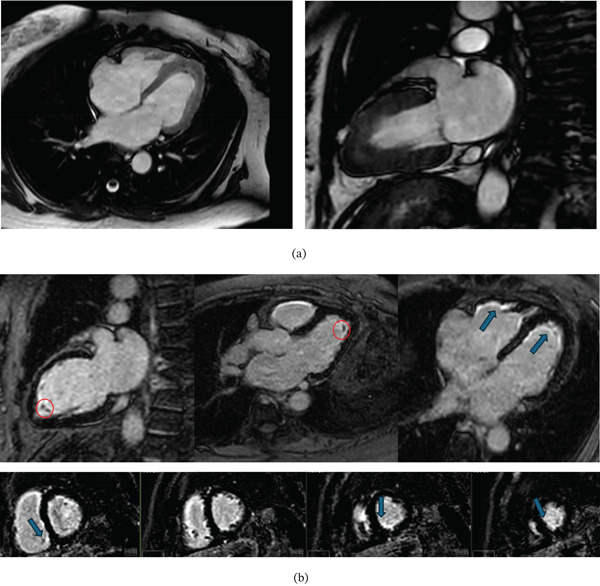
Axial view, cine showing apical bi‐ventricular increased apical wall thickness with obliteration of the left ventricular apex (a). Chamber view, cine showing apical increased apical wall thickness (b).

**Figure 3:**
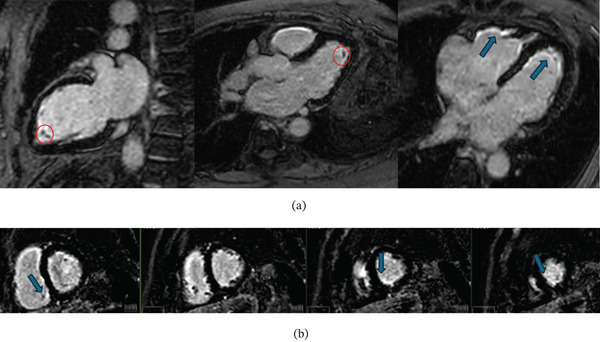
Late gadolinium enhancement inversion recovery images, 10 minutes after contrast injection, showing small left ventricular apical thrombus (red circles) and sub‐endocardial enhancement in left ventricle (LV) and right ventricle (RV) (blue arrows) (a). Short axis view shows sub‐endocardial enhancement in LV and RV (blue arrows) (b).

Echocardiography demonstrated a normal left ventricular ejection fraction (LVEF). ECG showed sinus bradycardia, anterior ST segment and T wave abnormalities with prolonged QT interval or TU fusion (Figure 1). Cardiac MRI revealed elevated T2 signals and an extracellular volume fraction of 32%, consistent with myocarditis and hypertrophic cardiomyopathy (Figures 2 and 3). Given severe thrombocytopenia (platelets 19 k/uL) and anemia (hemoglobin 8.9 g/dL), biopsy and left heart catheterization were not pursued. Laboratory testing confirmed positive coxsackievirus titers and marked eosinophilia—absolute eosinophils 2.96 k/uL, 34.9%.

## 5. Management

At discharge, the patient was prescribed prednisone 80 mg (for 2 weeks) and colchicine 1.2 mg daily, which initially improved her symptoms. However, within a month, she was readmitted due to worsening shortness of breath, ankle swelling, weight gain from edema, and NT‐proBNP of 2206 pg/mL. She was treated with furosemide 20 mg and metoprolol 25 mg. Workup for transthyretin (TTR) amyloidosis was negative. Despite treatment, she continued to experience congestive symptoms, including persistent shortness of breath and edema, which was managed in heart failure clinic.

## 6. Outcome and Follow‐Up

One month later, echocardiography revealed a right ventricular systolic pressure of 68 mmHg, and an estimated right atrial pressure of 15 mmHg based on inferior vena cava ultrasonography assessment. Despite treatment with furosemide and metolazone, the patient′s dizziness and shortness of breath worsened. Right heart catheterization showed normal right‐sided pressures but elevated pulmonary capillary wedge pressures (PCWP) and marginal cardiac output. The Holter monitor was negative, and NT‐proBNP remained elevated at 1802 pg/mL.

Repeat echocardiography demonstrated a preserved ejection fraction of 60*%* ± 5*%* but Grade III left ventricular diastolic dysfunction, suggestive of infiltrative cardiomyopathy with a restrictive filling pattern. Global longitudinal strain was significantly reduced (−8.4%). Right ventricular systolic function was moderately impaired, with a tricuspid annular displacement of 1.1 cm. Marked hypertrophy of the mid‐to‐distal right ventricular free wall and septum was noted, along with severe (3+ to 4+) tricuspid regurgitation secondary to annular involvement.

She was managed with metoprolol 25 mg and furosemide 40 mg daily along with metolazone 2.5 mg every other day and pulmonary hypertension was followed up with pulmonology.

## 7. Discussion

The clinical presentation of Löffler endocarditis can be diverse and nonspecific, often leading to misdiagnosis. [1] Patients may present symptoms such as chest pain, peripheral edema, and dyspnea, which can be indicative of heart failure. In this case, the patient exhibited these last three symptoms along with significant peripheral eosinophilia, which raised suspicion for direct eosinophilic cytotoxicity. Marked eosinophilia—absolute eosinophils 2.96 k/uL, 34.9%—along with echocardiographic findings, including right ventricular hypertrophy with a restrictive filling pattern and severe tricuspid regurgitation, supported the diagnosis of Löffler endocarditis.

Löffler endocarditis can cause restrictive cardiomyopathy due to eosinophilic infiltration of the endomyocardium leading to fibrosis and damage to atrioventricular valves. In Figures 2 and 3, elevated T2 signals and an extracellular volume fraction of 32% on cardiac MRI are consistent with myocarditis and hypertrophic cardiomyopathy. This restrictive pattern explained the presence of heart failure in this patient. Unfortunately, multiple attempts were made to get endomyocardial biopsy; however, only two samples were obtained, and both appeared to be scar tissue. The biopsy was difficult as the biotome was unable to produce premature ventricular contractions, likely due to subendomyocardial scar. The procedure was done with both echocardiogram and fluoroscopy guidance. Further biopsy attempts were aborted and the diagnosis of Löffler endocarditis was considered definitive given hypereosinophilia and strong evidence of cardiac damage.

The prevalence of cardiac involvement in adults with hypereosinophilia ranges from 40% to 50% [2]. This idiopathic form is part of the rare hypereosinophilic syndrome group, which is defined as eosinophilia (>1.5 × 10^9^/L) for more than 6 consecutive months associated with eosinophilic induced organ damage in the absence of hypereosinophilic causes such as allergic drug reactions, parasitic infections, or malignancy [3]. Clinical and laboratory features suggestive of a primary myeloid disorder include dysplastic eosinophils and eosinophil precursors in the blood, anemia or erythrocytosis, thrombocytopenia, splenomegaly, and elevated serum levels of vitamin B 12 and/or tryptase [4]. Although this patient had thrombocytopenia and anemia, the presence of cardiac damage was most compatible with the diagnosis of Löffler endocarditis.

The management of Löffler endocarditis primarily focuses on reducing eosinophilic infiltration and managing cardiac complications. Corticosteroids, such as high‐dose prednisone, are the mainstay of therapy due to their efficacy in suppressing eosinophilic activity and mitigating myocardial damage. In cases with limited response to glucocorticoids and eosinophilic granulomatosis with polyangiitis, additional immunosuppressive therapies may be considered, favoring cyclophosphamide or rituximab [5, 6]. Our patient initially responded to corticosteroid therapy; however, the recurrence of symptoms required further heart failure medical management. Surgical options include endomyocardial resection and valve replacement or repair, although data in patients with Löffler endocarditis are limited [5].

The progression of Löffler underlines the importance of early imaging diagnosis and intervention. As in this patient, advanced stages of the disease may lead to thrombosis development (Figure 3) and significant endomyocardial fibrosis with restrictive cardiomyopathy and atrioventricular valve damage. In this case, the small thrombus disappeared in following echocardiograms without anticoagulation due to the patient′s low platelet count. Nevertheless, clinicians should maintain a high index of suspicion for Löffler endocarditis in patients presenting with evidence of infiltrative cardiomyopathy on MRI, unexplained heart failure, and hypereosinophilia [7, 8]. Early recognition and appropriate management are important to improve outcomes in this rare but potentially severe condition.

## 8. Conclusions

Löffler endocarditis is a rare condition, and patients can present with a wide range of nonspecific symptoms, making diagnosis challenging. This case highlights the importance of cardiac MRI in identifying endomyocardial damage in patients with unexplained heart failure and peripheral eosinophilia. Although early recognition and intervention can be challenging, they are necessary in managing Löffler endocarditis. Prompt initiation of corticosteroid therapy can mitigate eosinophilic inflammation and prevent further damage while assessing potential underlying causes of hypereosinophilia.

## Funding

This research was funded by the Heart, Vascular and Thoracic Institute.

## Consent

Written informed consent has been obtained.

## Conflicts of Interest

The authors declare no conflicts of interest.

## Data Availability

The data that support the findings of this study are available on request from the corresponding author. The data are not publicly available due to privacy or ethical restrictions.
